# Lactotransferrin expression is downregulated and affects the mitogen-activated protein kinase pathway in gastric cancer

**DOI:** 10.3892/ol.2021.12504

**Published:** 2021-02-01

**Authors:** Gengqiu Luo, Yanhong Zhou, Wei Yi, Hong Yi

Oncol Lett 9: 2409-2413, 2015; DOI: 10.3892/ol.2015.3011

Following the publication of the above article, an interested reader drew to the authors’ attention that, in Fig. 4 on p. 2412, the data intended to show the p38 and JNK2 protein bands were apparently the same. The authors re-examined their original data, and realized that the JNK2 data had inadvertently been inserted into the Figure twice.

The corrected version of Fig. 4, showing the correct data for the p38 protein bands, is shown opposite. Note that the correction made to this figure does not affect the overall conclusions reported in the paper. The authors are grateful to the Editor of *Oncology Letters* for granting them the opportunity to publish this corrigendum, and apologize to the readership for any inconvenience caused

## Figures and Tables

**Figure 4.**
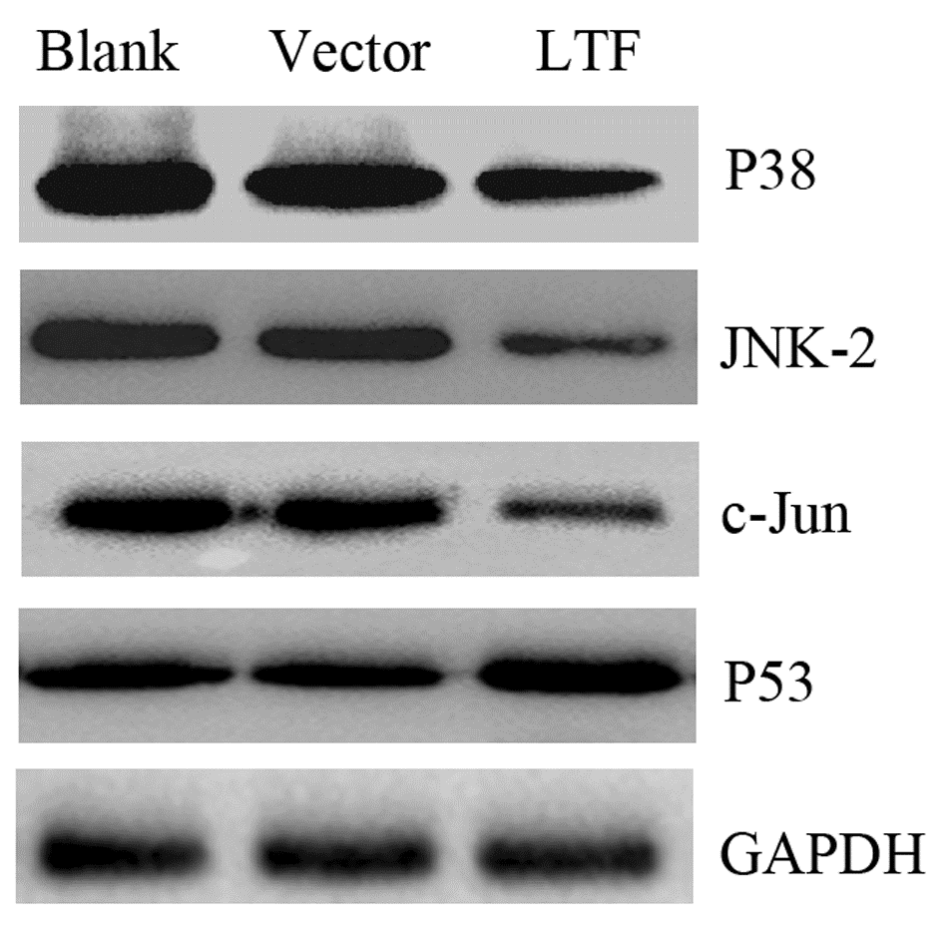
Protein expression levels of p38, JNK2, c-Jun and p53 in the gastric cancer cell line, SGC7901. LTF, SGC7901 cells transfected with pIRES-LTF plasmid; vector, SGC7901 cells transfected with pIRES plasmid; blank, untransfected SGC7901 cells. Data are representative of three independent experiments. JNK, c-Jun N-terminal kinase; LTF, lactotransferrin.

